# Physiological response to weight carrying and associations with conformation traits in Icelandic horses used for tour riding

**DOI:** 10.1186/s13028-025-00818-5

**Published:** 2025-06-19

**Authors:** Denise Söderroos, Guðrún Jóhanna Stefánsdóttir, Sveinn Ragnarsson, Víkingur Gunnarsson, Anna Jansson

**Affiliations:** 1 Department of Animal Biosciences, Faculty of Veterinary Medicine and Animal Science, Swedish University of Agricultural Sciences, 750 07 Uppsala, Sweden; 2 Department of Equine Science, Hólar University, 551 Sauðárkrókur, Iceland

**Keywords:** Back, Body measurements, Equine, Rider weight, Weight carrying exercise test

## Abstract

**Background:**

Weight carrying capacity is an important trait in riding horses and it may be associated with conformation. This study examined the physiological response to a ridden incremental weight carrying test in 16 adult Icelandic horses used for tour riding. Horses carried 20% (BWR_20%_), 25% (BWR_25%_), 30% (BWR_30%_) and 35% (BWR_35%_) of their body weight (BW) in tölt (~ 5.7 m/seconds, 640 m/step), and associations with body measurements and back conformation (score) were examined. Horses were divided into two groups (narrow or broad back) and body measurements were collected. Plasma lactate was analysed in blood samples collected after each step in the exercise test, an exponential equation was fitted, and BW-ratio was calculated for 2, 3 and 4 mmol/L (BWR_La2_, BWR_La3_ and BWR_La4_). Plasma creatine kinase (CK) and aspartate amino transferase (AST) were analysed at rest and 24 h post exercise.

**Results:**

Four out of 15 horses did not reach a plasma lactate concentration of 4 mmol/L, even at BWR_35%_. A positive correlation was found between chest width and BWR_La4_ and between the difference between height at withers and croup and BWR_La2_ (P < 0.05). Hock circumference and the difference between height at croup and back were negatively correlated with BWR_La2_ (P < 0.05). The change in CK from rest to 24 h post exercise was negatively correlated with the difference between height at withers and height at back and croup (P < 0.05).

**Conclusions:**

The physiological response to weight carrying was relatively low. A wider chest, “uphill” conformation, straight backline and smaller hock circumference were associated with weight carrying capacity, but group (narrow or broad back) was not.

**Supplementary Information:**

The online version contains supplementary material available at 10.1186/s13028-025-00818-5.

## Background

The Icelandic horse is a popular breed in numerous countries that is often used for tour riding and in riding schools. The breed is rather small, with mean height at withers around 140 cm and body weight (BW) 330–370 kg [1]. Compared with other breeds of similar size, the Icelandic horse is more often ridden by adult riders, creating a need for great weight carrying capacity in the breed. It is well known that increased weight carrying during exercise affects the physiological response [2–4] and gait parameters [5–7] in several horse breeds, including the Icelandic horse. Increased weight carrying might also influence the behaviour [7]. Some tour riding companies and equestrian associations have implemented weight limits of the rider intended to enhance the welfare of the horses [4, 8]. However, scientific knowledge about weight carrying capacity and associations with conformation is needed and could be useful e.g. in riding schools and in tour riding companies.

A previous study investigated the physiological response during a weight carrying exercise test in Icelandic horses used for higher riding education [4]. In that study, BW-ratio (BWR) between rider and horse was 20, 25, 30 and 35% and showed a positive correlation with magnitude of the physiological response in the horses. On average, the horses reached the lactate threshold (plasma lactate concentration 4 mmol/L) at BWR ~ 23%, but horses with higher back score (i.e. a broader back at the thoracolumbar region) appeared to reach the lactate threshold at higher BWR [4]. Similarly, another study [3] found that light riding horses with wider loins (measured at the cross-section between L1 and L2 vertebra) showed less muscle soreness and tightness 24 h after weight carrying compared with horses with narrower loins. A broad, well-muscled back is also anecdotally perceived to be beneficial for weight carrying capacity in horses and is rewarded in subjective conformation assessments in breed evaluation field tests (BEFTs) for Icelandic horses [9]. A recent study [10], which subjectively assessed weight carrying capacity in 65 Icelandic horses used for tour riding and in riding schools found that weight carrying capacity was positively correlated with height at withers, back and croup, but not with the difference between height at withers and height at back and croup. A greater difference between height at withers and height at back and croup (“uphill” conformation) is known to be important for riding ability in Icelandic horses participating in BEFTs [11]. Studies investigating the influence of conformation on weight carrying capacity are however scarce and the findings are not entirely consistent [3, 4, 10]. Further knowledge about this, and especially if it can be applied to individual horses, could be useful when matching riders with horses, and thus for animal welfare and performance.

The aims of the present study were to examine the physiological response to a ridden weight carrying exercise test in Icelandic horses used for tour riding and (i) associations with body measurements and (ii) the effect of a narrow and a broad back. It was hypothesised that a broad back is associated with improved weight carrying capacity.

## Methods

### Horses

The experimental study was conducted at Hólar University, Iceland, on two occasions in October 2021 and 2022. In total 16 horses (nine geldings, seven mares), aged 10–22 years and with BW 386 ± 32 kg recorded 1–3 days before the exercise test, were included in the analysis of the association between physiological response at a ridden weight carrying exercise test and body measurements. Prior to the study, horses were selected during visits to two tour riding companies (n = 4 and 12 horses, respectively), located near the university. All horses had been used in tours during the previous summer and occasionally during September. All were assessed by their owners as able to perform the planned ridden weight carrying exercise test. Half of the horses from each company were scored with a back score of < 4.5 (median 4.25), i.e. narrow back (N) and the other half, with the same work experience and of equal age, with a back score of > 4.5 (median 4.9), i.e. broad back (B) [4, 12], resulting in two different groups (Table 1). Back score has been shown not to be significantly affected by changes in overall body condition (scores 5.7–6.1) of Icelandic horses [13]. At the time of the experiment, back was scored lower (a score of 4.5) than in pre-selection in two horses initially included in group B. Therefore, these two horses were excluded from analysis of the effect of a narrow or broad back, which resulted in a total of 14 horses (N: n = 8, B: n = 6) included in that analysis (Table 1). The number of horses included in each group was determined from a power calculation based on results from a previous study [4].

**Table 1:** Back measurements, BCS^a^ and age (mean ± SD) of 14 participating Icelandic horses

Variable	N^b^	B^c^	*P*-value
Back score^d^	4.25 (range 4.0–4.25)	4.90 (range 4.75–6.0)	< 0.05
Back angle (degrees)^e^	18.3 ± 1.0	14.5 ± 1.4	< 0.0001
Loin angle (degrees)^f^	16.5 ± 1.7	13.4 ± 1.1	< 0.05
BCS^a^	5.1 ± 0.5	6.2 ± 0.4	< 0.05
Age (years)	15 ± 4	15 ± 3	0.713

*BCS* body condition score

^a^Assessed on a continuous scale of 1–9, including assessments of ribs, back and tail head [12]

^b^Horses with narrow back (n = 8)

^c^Horses with broad back (n = 6)

^d^Values shown are median, due to non-normally distributed data

^e^Measured with a flexible curve ruler placed at the 18th thoracic vertebra

^f^Measured with a flexible curve ruler placed at the lumbar region at the level of the caudal part of the last rib

### Preparation

The selected horses arrived at the university 9–12 days before the exercise test for acclimation and training (10 in 2021, six in 2022), except for two horses that arrived five and six days before the exercise test, respectively. The horses were housed in individual boxes and kept outside in a gravel paddock for 0–3 h/day and on pasture for 1–3 h/day. On the experimental day, the horses were kept in their boxes until the exercise test. At arrival and at least one more time during the preparation period, the horses were weighed (Tru-Test scales, Model 702; Tru-Test Ltd, Auckland, New Zealand) in order to adjust the amount of feed. In addition to pasture, hay was fed in the boxes three times/day (in total 0.75–1.0 kg dry matter (DM)/100 kg BW/day, with net energy and crude protein content 4.7 ± 0.8 MJ/kg and 155 ± 26 g/kg DM, respectively), based on recommendations of the minimum forage intake [14]. All horses had free access to salt blocks and water from automatic water bowls in the boxes. No feed was offered 2–3 h before the exercise test. All horses were ridden (by the same rider as in the exercise test) at least once during the preparation period and they were also exercised on a dry high-speed treadmill 4–6 times in walk and trot (< 30 min (min) at sub-maximal intensity, heart rate (HR) < 210 beats per minute (bpm)). All were rested for at least one day before the exercise test. To exclude any unhealthy and/or lame horses and evaluate if the exercise test had a negative impact on the health, a clinical examination was performed before the exercise test and 24 h after the test, by the same veterinarian who was blinded to groups. The examination included palpation of all four limbs and the thoracolumbosacral region, auscultation of the heart, using a stethoscope, and a flexion test of the limbs (including both proximal and distal joints) with a trot-up for approximately 50 m on gravel (all horses were shod).

### Body measurements

Body condition score, angle of the thoracolumbar region and body measurements were collected.

**Fig. 1:**
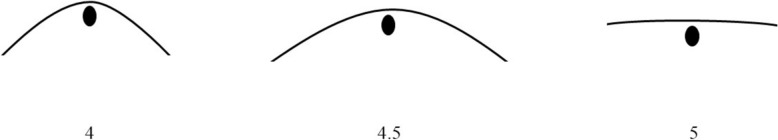
Illustration of the thoracolumbar region of the back with different condition scores. Scores are ranging from 4 to 5 with 0.25 increments (a score of 4.25 and 4.75 are not shown in the figure) as described in a previous study [4] and based on the Henneke scale [12]. The backs are illustrated from a caudal view, and the black dots symbolises the spine

**Fig. 2:**
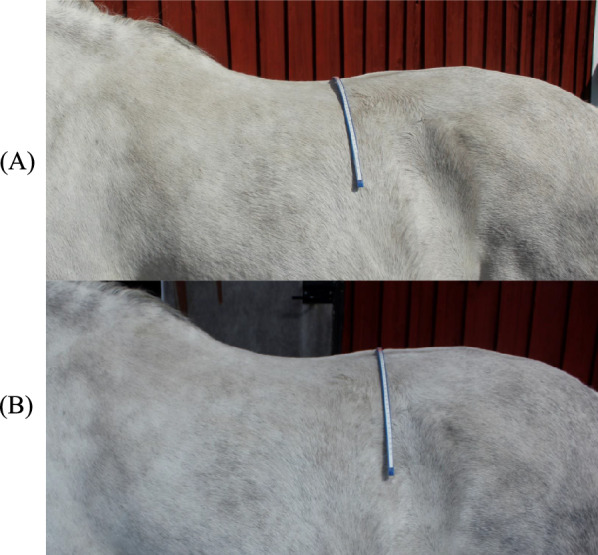
Measurement of the thoracolumbar region at two different sites using a flexible curve ruler. Flexible curve ruler placed at (**a**) the 18th thoracic vertebra identified by palpation of the last rib (i.e. back angle) and (**b**) at the height of the caudal part of the last rib (by palpating vertically from the rib up to the dorsal aspect of the lumbar region) (i.e. loin angle)

**Fig. 3:**
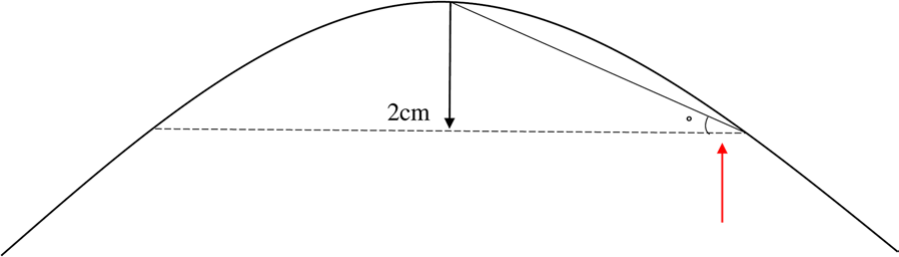
Curve from measurements of the thoracolumbar region on Icelandic horses using a flexible curve ruler. The angle (red arrow) of the region was measured from the drawn curve, using a goniometer [10]

Body condition score in the horses was assessed using a modified version of the Henneke scale [12, 15], where a separate score for ribs, thoracolumbar region (i.e. the back) and tail head was given on a continuous scale of 1–9. The overall BCS was calculated as a mean value from the three different body parts. The back was scored with an accuracy of 0.25 (Fig. 1), as described in a previous study [4]. The angle of the thoracolumbar region was measured by forming a flexible curve ruler (60 cm; Donau Elektronik GmbH, Matten, Germany) transversely over the back of the horse at two different sites: (i) at the 18th thoracic vertebra identified by palpation of the last rib (i.e. back angle), and (ii) at the height of the caudal part of the last rib (by palpating vertically from the rib up to the dorsal aspect of the lumbar region, i.e. loin angle; Fig. 2). The centre of the ruler was marked to ensure accurate positioning on the vertebra. The ruler was then removed carefully and placed onto a paper and the resulting curve was drawn. A horizontal line, extending from the left to the right side of the curve, was drawn 2 cm below the top of the curve. The angle between the top and the left and right side of the curve, at the level of the drawn line, was measured with a goniometer (15 cm; Primed, Halmstad, Sweden; Fig. 3) and a mean value was calculated from both sides of the curve. The within coefficient of variation (CV) for measurements of the angle at the 18th thoracic vertebra using the flexible curve ruler was in a previous study 1.2%, based on duplicate measurements on 68 horses [10]. The angle measurements were performed by this same person in the current study.

**Fig. 4:**
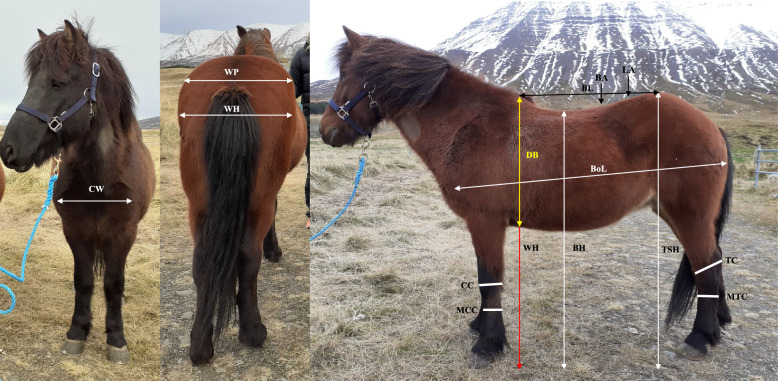
Body measurements collected in 16 Icelandic horses. The measurements were: height at withers (highest thoracic vertebra) (yellow + red arrow) (WH), height at the lowest point of the back (BH), height at the highest point of the croup (*tubera sacrale*) (TSH), back length (BL), measured from the cranial part of *tubera sacrale* to the highest point of the withers, body length (BoL), measured from *tuberculum majus humeri* to the caudal end of the hindquarters (horizontally to *tuber ischiadicum*), breast depth (DB) (yellow arrow), measured from the withers and straight down to *sternum*, chest width (CW), measured between the points of the shoulders (*tuberculum majus humeri*), width of the *pelvis* between the points of the *pelvis* (*tubera coxae*) (WP), width of the hips between the hip joints (WH), maximum circumference of *carpus* (CC) and hock (*tarsus*) (TC) and minimum circumference of *metacarpus* (MCC) and *metatarsus* (MTC). Notice that the figure only describes the measurements and not the experimental conditions (experimental measurements were performed indoors on concrete floor and with horses in even body position)

Height at the highest point of the withers (highest thoracic vertebra), height at the lowest point of the back, height at the highest point of the croup (*tubera sacrale*), body length and breast depth were measured with a measuring stick. Body length was measured from the point of the shoulder (*tuberculum majus humeri*) to the caudal end of the hindquarters (horizontally to *tuber ischiadicum*) and breast depth was measured from the withers and straight down to the breastbone (*sternum*). Width of the chest between the points of the shoulders (*tuberculum majus humeri*), width of the *pelvis* between the points of the *pelvis* (*tubera coxae*), and width of the hips between the hip joints (*articulatio coxae*) were measured with a calliper. The maximum circumference of *carpus* and hock (*tarsus*), the minimum circumference of *metacarpus* and *metatarsus*, and back length were measured with a measuring tape. The circumference of the hock was measured around *tuber calcanei* and at the angle of the distal hock joints, and back length was measured from the cranial part of *tubera sacrale* to the highest point of the withers (Fig. 4).

All body measurements were made one or two days before the exercise test, with the horse standing square on a concrete floor, by the same person and at approximately the same time during the day for all horses (Table 2).

**Table 2:** Body measurements collected in 16 Icelandic horses

Variable (cm)	Mean ± SD	Range
Height at withers	138 ± 4	134–144
Height at back	127 ± 3	122–131
Height at croup (*tubera sacrale*)	136 ± 3	131–142
Height at withers–Height at back	11 ± 2	8–14
Height at withers–Height at croup (*tubera sacrale*)	3 ± 2	0–8
Height at croup (*tubera sacrale*)–Height at back	9 ± 2	4–11
Back length^a^	77 ± 3	71–83
Body length^b^	145 ± 3	141–153
Breast depth^c^	66 ± 2	62–70
Width of the chest^d^	37 ± 2	33–39
Width of the *pelvis*^e^	49 ± 2	45–53
Width of the hips^f^	45 ± 2	43–49
Circumference of *carpus*^g^	29 ± 1	27–31
Circumference of *metacarpus*^h^	19 ± 1	18–21
Circumference of hock^g^	36 ± 1	34–38
Circumference of *metatarsus*^h^	21 ± 3	18–31
Format of the horse^i^	7 ± 3	1–15

^a^Measured from the cranial part of *tubera sacrale* to the highest point of the withers

^b^Measured from *tuberculum majus humeri* to the caudal end of the hindquarters (horizontally to *tuber ischiadicum*)

^c^Measured from the withers and straight down to *sternum*

^d^Measured between the points of the shoulders (*tuberculum majus humeri*)

^e^Measured between the points of the *pelvis* (*tubera coxae*)

^f^Measured between the hip joints (*articulatio coxae*)

^g^Maximum circumference

^h^Minimum circumference

^i^Difference between body length and height at withers

### Exercise test

A ridden incremental weight carrying exercise test [4], was performed outdoors on a 257 m oval gravel track during two (2021) or three (2022) consecutive days. Equal number of horses from each group (N or B) performed the test in 2021 and 2022, to limit the effect of year. Horses from each group performed the test in alternating order, to limit effects of e.g. time of the day and weather conditions. In 2021, one male rider (73 kg, 178 cm) rode all horses, while in 2022, one female rider (70 kg, 165 cm) rode all horses. Both were advanced riders with a BSc degree in riding and riding teaching from Hólar University. The exercise test (total distance 2.9 ± 0.1 km) consisted of four steps with increasing weight carried by the horse for each step (see below) and 2.5 coherent laps on the track were ridden for each step. The horses carried 20.5 ± 1.4% of their BW in step 1 (BWR_20%_), 25.3 ± 0.4% in step 2 (BWR_25%_), 30.3 ± 0.3% in step 3 (BWR_30%_) and 35.4 ± 0.7% in step 4 (BWR_35%_). The weight carried in the exercise test was based on BW measured 1–3 days before the exercise test. The test was ridden on the left hand in tölt, at an average speed of 5.7 ± 0.2 m/seconds (s), which did not differ between the groups (P > 0.05). To facilitate adjustment of the speed, one person recorded the time between markings on the track, using a stopwatch, and regularly informed the rider via wireless communication (earpiece) if the intended speed (~ 5.5 m/s, i.e. similar to the mean speed measured in a previous study [4]) was maintained.

The exercise test was preceded by a mounted warm-up (total distance 1.6 ± 0.1 km) carrying the same weight as in step 1. The warm-up consisted of 5–7 min walking (1.8 ± 0.2 m/s) and 1.1 ± 0.1 km in tölt (4.9 ± 0.3 m/s). After the warm-up and between each step, horses were stopped and the rider dismounted for 7.0 ± 2.6 min to add weights and for collection of a blood sample.

Two different saddles were used (weight 6.0 kg and 8.4 kg, respectively), depending on the intended weight load. The heavier saddle (Ástund Super; Ástund, Reykjavík, Iceland) was specially adapted (by the producing company Ástund) with bags sewn onto the flaps where it was possible to add lead weights up to 8 kg on each side. In addition, two different saddle pads (weight 2.0 kg and 13.5 kg, respectively), extra-heavy stirrups (4 kg each) and a vest designed for scuba diving where lead weights (up to 16 kg) could be added to pockets. Due to discomfort for the rider, a weight vest specially designed for CrossFit^®^ was used instead of the scuba diving vest during the experiment in 2022. To avoid imbalance, weights were added evenly on both sides. For the three horses with the greatest BW, during step 4 (BWR_35%)_ a backpack filled with sand (up to 16 kg) was carried by the rider, either on the back or in front, in order to reach the intended weight. In 2022, two horses from group N and one horse from group B had boots on the front hooves (155–170 g each) in order to keep a clear-beat tölt during the exercise test.

Ambient temperature and wind speed were recorded continually at an automatic weather station (Model WH-1080; Clas Ohlson, Insjön, Sweden) located ~ 100 m from the riding track, and did not differ between years (P > 0.05). Values when the horse left the stable for the exercise test were noted. In the middle of each experimental day (10.00–13.00), median ambient temperature was 5.4 °C (range − 4.7–6.3 °C) and median wind speed was 2.7 m/s (range 0–8 m/s). Data on of wind speed were missing for one day, but it was not unusually windy on that day.

### Data collection

Heart rate, speed and distance were recorded continuously in the horses, using a HR recorder and GPS device (Polar Vantage M, HR Monitor; Polar Electro Oy, Kempele, Finland) from before the horses left the stable to 5 min after the exercise test. According to the manufacturer’s technical specification, the accuracy of measured speed was ± 2 km/h and of measured distance ± 2%. The HR recorder and GPS device had a sampling rate of one measurement/s. Data from the HR recorder were imported into Microsoft Excel 2016 (Microsoft, Redmond, WA, USA) and a mean HR-value for the last 15 s in each step of the exercise test was calculated. During rest (before leaving the stable) and after 5 min of recovery, HR was noted directly in the HR recorder. Respiratory rate (RR) was measured by counting number of breaths for 15 s during rest and after 5 min of recovery. Rectal temperature (RT) was measured at rest and after a 5 min recovery, using a digital thermometer (Omron; HealthCare, Europe).

### Blood sampling and analysis

Blood samples were collected in lithium heparin tubes (BD Vacutainer^®^, Plymouth, UK) at rest (2–6 days before the exercise test), immediately after warm-up and each step in the exercise test and after 5 min of recovery. Local anaesthesia was given before venipuncture (20 mg/mL, Xylocaine; AstraZeneca, Södertälje, Sweden). After collection, the blood samples were directly placed on ice. Hematocrit was measured (within one hour) in all samples except those collected at rest and after warm-up in 2022, using capillary glass tubes and centrifugation (8 min, 21,913 × g, Cellokrit; AB Lars Ljungberg & Co, Stockholm, Sweden). Duplicate analyses were performed, and the mean value was used in statistical analysis. To ensure that all horses reached, or were close to, the lactate threshold during the exercise test, blood lactate concentration was analysed directly after the exercise test in the samples collected after step 4 (Lactate Pro 2 analyser; Arkray, Kyoto, Japan). Horses with low (< ~ 3 mmol/L) blood lactate concentrations were re-tested two days later (n = 2) with higher final BWR, and data on physiological parameters from the second exercise test were used in statistical analysis. Results from the Lactate Pro 2 analyser were not reported or used in the statistical analysis (see plasma lactate below).

Plasma was separated by centrifugation (10 min, 3000 rounds/min; Hettich, Tuttlingen, Germany) and stored at − 18 °C until further analysis. Plasma lactate was analysed by the laboratory at the Swedish University of Agricultural Sciences (SLU), in duplicate with an YSI 2500 Biochemistry Analyzer (YSI, Yellow Springs, Ohio, USA, CV = 1.6%), and a mean value was used for statistical analysis. Aspartate amino transferase (AST) and creatine kinase (CK) were analysed by SLU University Animal Hospital, in duplicate with a Chemistry Analyser (DxC 700 AU; Beckman Coulter, Indianapolis, USA, CV for AST = 0.3% and CK = 0.6%) in samples collected 2–6 days before the exercise test and ~ 24 h after the exercise test.

### Statistical analysis and calculations

Body weight ratio at a plasma lactate concentration of 2 mmol/L (BWR_La2_), 3 mmol/L (BWR_La3_) and 4 mmol/L (BWR_La4_) were estimated for individual horses in Microsoft Excel version 2016 (Microsoft, Redmond, Washington, USA) by obtaining the equation of the exponential regression between BWR and plasma lactate concentration for each step in the exercise test (Additional file 1). In one horse, a substantial part of step 4 was performed in gallop (and at a higher speed than the other steps). In another horse, the lactate threshold was reached already before the first step and therefore, BWR_La2_, BWR_La3_ and BWR_La4_ were not estimated for these two horses. The BWR when the horse reached a HR of 180 bpm (BWR_180_) and 190 bpm (BWR_190_) were estimated for individual horses in Microsoft Excel version 2016, by obtaining the equation of the linear relationship between BWR for each step and the mean HR of the last 15 s in each step (Additional file 1). For one horse, HR data were lost in steps 2 and 3 due to connection issues with the HR recorder, while for another horse, there was no linear increase in HR, and these data were omitted from the analysis. In addition to BWR_180_ and BWR_190_, BWR_200_ was estimated initially, but was not included in the final analysis due to few observations (i.e. the majority did not reach a HR of 200 bpm).

Statistical analyses were performed using SAS (version 9.4; SAS Institute Inc., Cary NC, USA). The effect of group on BWR_La2_, BWR_La3_, BWR_La4_ and physiological parameters in individual samples was analysed with a general linear model (PROC GLM) using the following model (i): Y = µ + a_i_ + b_j_ + c_k_ + d_l_ + e_ijkl_ where Y is observation, µ is mean value, a_i_ is fixed effect of group, b_j_ is fixed effect of year, c_k_ is fixed effect of sex, d_l_ is continuous effect of BCS and e_ijkl_ is the residuals (see statistical code in Additional file 1).

When several samples were included in the analysis, the effects of group and sample on physiological parameters were analysed with a general linear mixed model (PROC MIXED), adjusted for repeated measurements, and including the same fixed effects as in model (i), the interaction effect between group and sample and horse as a random effect. Speed was included as a continuous effect when analysing HR data, but was excluded from the other analyses since it had no effect in those (P > 0.05). Ambient temperature and wind speed were initially included in model (i) when analysing RR and RT after 5 min of recovery and BWR was initially included in the statistical model as a continuous effect when analysing physiological parameters. However, they had no effect (P > 0.05) and were therefore excluded from the final model.

Since several horses did not reach the lactate threshold, the horses were divided into two groups based on whether they reached the lactate threshold or not during the weight carrying exercise test. To investigate possible differences in body measurements between these groups, the following general linear model (PROC GLM) was applied (ii): Y = µ + a_i_ + b_j_ + c_k_ + e_ijk_, where Y is observation, µ is mean value, a_i_ is fixed effect of group, b_j_ is fixed effect of sex, c_k_ is continuous effect of age and e_ijk_ is the residuals (see statistical code in Additional file 1).

A *t*-test was used to compare weather conditions between years and the angle of the thoracolumbar region, BCS, age, BWR for each step in the exercise test and physiological parameters at rest between groups. Comparison of back score between groups was performed with a Wilcoxon rank sum test. Possible group effects on speed measured by the HR recorder were analysed with a general linear mixed model (PROC MIXED), adjusted for repeated measurements (iii): Y = µ + a_i_ + b_j_ + c_k_ + e_ijk_ where Y is observation, µ is mean value, a_i_ is fixed effect of group (N or B), b_j_ is fixed effect of sample, c_k_ is random effect of horse and e_ijk_ is the residuals (see statistical code in Additional file 1). Year (2021 or 2022) was initially included in model (iii) but was not significant and was removed from the final model.

Data on physiological parameters are presented as least square mean (LSM) ± standard error (SE) and values for BWR_180_, BWR_190_ and BWR_La4_ when including all horses and other parameters as mean ± standard deviation (SD), unless anything else is stated. Correlations between body measurements and physiological parameters (BWR_180_, BWR_190_, BWR_La2_, BWR_La3_, BWR_La4_ and the difference in AST and CK levels between rest and 24 h post exercise) were determined using Pearson’s correlation test. Normality of residuals was confirmed by visual assessments of QQ-plots. The significance level was set at P < 0.05.

## Results

All horses except three completed the study with no signs of injury. Two horses (one from each group) had minor oedema in the distal limbs one day after the exercise test, and one of them (from group N) also had a small wound on the heel bulb at the left forelimb. Both these horses reached or exceeded the lactate threshold during the exercise test. Another horse, from group N, showed mild soreness on one side of the thoracolumbar region on the day after the exercise test. That horse also reached the lactate threshold during the exercise test. Plasma AST was higher before exercise (8.7 ± 0.4 µkat/L) compared with 24 h after the exercise test (8.2 ± 0.4 µkat/L; P < 0.05), but plasma CK did not differ between these time points (P > 0.05). In the horse with mild soreness, post exercise plasma AST (7.2 µkat/L) was near the median (7.1 µkat/L, range 6.1–16.4 µkat/L). A similar pattern was observed for CK (5.5 µkat/L, compared with median 6.6 µkat/L, range 3.8–25.3 µkat/L).

### Physiological responses to the weight carrying exercise test

Four out of 15 horses (excluding horse 16, which performed a substantial part of step 4 in gallop) did not reach the lactate threshold, i.e. not even at BWR_35%_, and thus BWR_La4_ could not be estimated. One horse reached the lactate threshold already in step 1 and another six horses had plasma lactate concentration > 2 mmol/L in step 1 (and were therefore excluded from the analysis of BWR_La2_ and BWR_La3_). It was possible to estimate BWR_180_ and BWR_190_ for a total of five horses, respectively, since the other horses did not reach these HR values (n = 5) or had higher HR already in step 1 (n = 9). Mean BWR_180_ and BWR_190_ was 26.2 ± 3.4% and 26.8 ± 4.7%, respectively.

### Response in all horses, irrespective of group

**Fig. 5:**
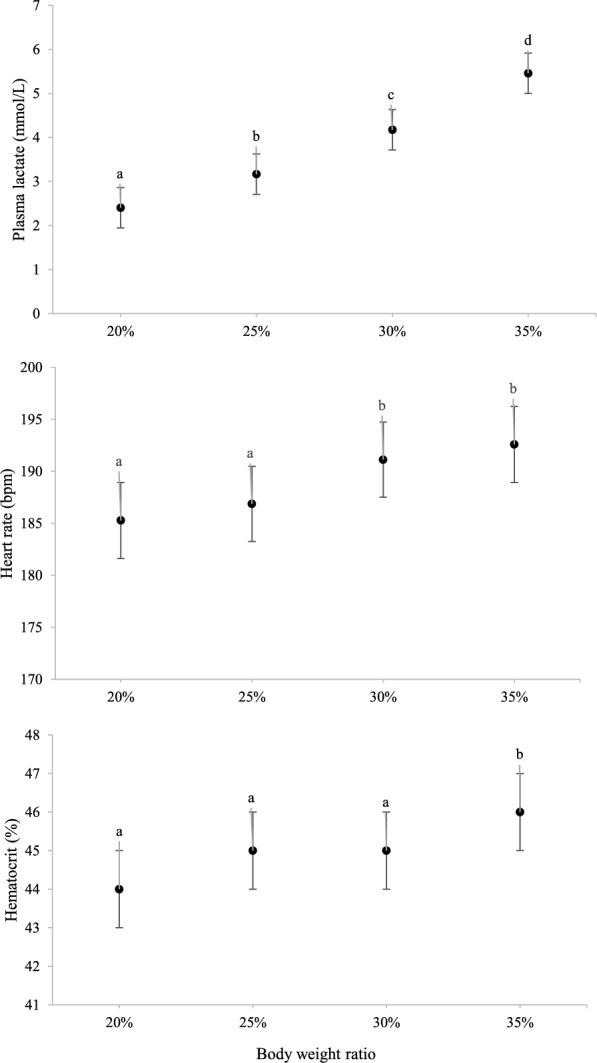
Physiological responses recorded in 14 Icelandic horses during a ridden exercise test. Horses carried 20, 25, 30 and 35% of their body weight (LSM ± SE). Different letters (**a**–**d**) indicate differences between body weight ratio values (P < 0.05)

Plasma lactate concentration (n = 14 horses) increased (P < 0.05) for each step in the exercise test (Fig. 5) and BWR_La4_ was 31.4 ± 4.1% (range 24.6–36.5%, n = 10 horses). Heart rate (n = 14 horses) was higher (P < 0.05) at BWR_30%_ and BWR_35%_ compared with BWR_20%_ and BWR_25%_ (Fig. 5, P < 0.05). Hematocrit (n = 14 horses) was higher (P < 0.05) in BWR_35%_ than in the other steps (Fig. 5). Respiratory rate and RT (n = 14 horses) were higher (P < 0.0001) after 5 min of recovery (82 ± 4 breaths/min and 38.5 ± 0.1 °C, respectively) than before exercise (30 ± 4 breaths/min and 37.0 ± 0.1 °C, respectively).

### Group responses

Body weight ratio at plasma lactate concentration of 2 mmol/L, BWR_La3_ and BWR_La4_ did not differ between the groups N and B (Table 3, P > 0.05). Plasma lactate concentration and hematocrit also did not differ between the groups (P > 0.05), based on samples from all steps (1–4) during the exercise test or individual samples. However, for the hematocrit there was a significant interaction (P < 0.05), and in group B, the hematocrit was higher (P < 0.05) in BWR_35%_ than in BWR_20%_ and BWR_25%_, and this was not observed in group N (P > 0.05). Also, hematocrit was higher (P < 0.05) in BWR_30%_ than in BWR_20%_ in group B. The overall HR during the exercise test (recorded during steps 1–4) and in individual samples did not differ between the groups (P > 0.05). Respiratory rate and RT recorded at rest and after 5 min of recovery also did not differ between the groups (Table 3, P > 0.05). Group had no effect on CK and AST levels or on the change in AST and CK levels from rest to 24 h post exercise test (Table 3, P > 0.05).

**Table 3:** Physiological responses in Icelandic horses during an incremental weight carrying test (LSM ± SE)

Physiological parameter	n	N^a^	B^b^	*P*-value
BWR_La2_^c^ (%)	8	27.4 ± 5.1	21.4 ± 6.9	0.642
BWR_La3_^c^ (%)	12	29.3 ± 2.6	26.5 ± 3.5	0.606
BWR_La4_^c^ (%)	10	31.3 ± 2.5	29.7 ± 2.9	0.734
Hematocrit_exercise_^d^ (%)	14	45 ± 1	44 ± 0.9	0.581
Hematocrit_step 4_	14	46 ± 1	46 ± 1	0.642
RR_recovery_^e^ (breaths/min)	14	77 ± 8	86 ± 10	0.570
RT_recovery_^e^ (°C)	14	38.7 ± 0.2	38.2 ± 0.2	0.121
CK_24h_^f^ (µkat/L)	14	7.6 ± 1.1	7.1 ± 1.4	0.796
AST_24h_^f^ (µkat/L)	14	8.9 ± 0.7	7.5 ± 0.9	0.321
CK_diff_^g^ (µkat/L)	14	0.2 ± 0.7	1.6 ± 0.9	0.337
AST_diff_^g^ (µkat/L)	14	− 0.6 ± 0.4	− 0.3 ± 0.5	0.321

*LSM* least square mean

^a^Horses with narrow back (n = 8), i.e. a back score < 4.5 (median 4.25) [12]

^b^Horses with broad back (n = 6), i.e. a back score > 4.5 (median 4.90) [12]

^c^Body weight ratio at plasma lactate concentration of 2, 3 and 4 mmol/L

^d^Average hematocrit including all samples from the exercise test (steps 1–4)

^e^Respiratory rate (RR) and rectal temperature (RT) recorded 5 min post exercise

^f^Plasma creatine kinase (CK) and aspartate amino transferase (AST) levels recorded 24 h post exercise

^g^Difference between levels at rest and 24 h post exercise (post exercise minus rest)

### Correlations between physiological responses and body measurements

**Fig. 6:**
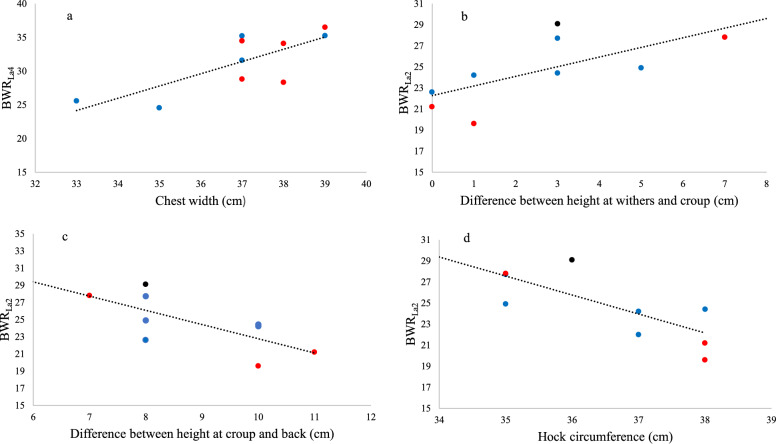
Relationships between physiological parameters and body measurements in Icelandic horses performing a weight carrying test. The figure shows the relationship between **a** body weight ratio (BWR) at the lactate threshold (BWR_La4_) and chest width (r = 0.77, P < 0.05), and between BWR at a plasma lactate concentration of 2 mmol/L (BWR_La2_) and **b** the difference between height at withers and height at croup (*tubera sacrale*) (r = 0.68, P < 0.05), **c** the difference between height at croup (*tubera sacrale*) and height at back (r = − 0.71, P < 0.05) and **d** hock circumference (r = − 0.77, P < 0.05). Blue dots represent horses with narrow back (back score < 4.5), red dots horses with broad back (back score > 4.5) and black dots horses with a back score of 4.5 [12]

Chest width was positively correlated (r = 0.77, P < 0.05) with BWR_La4_ (Fig. 6). Body weight ratio at plasma lactate concentration of 2 mmol/L was positively correlated (r = 0.68, P < 0.05) with the difference between height at withers and height at croup (*tubera sacrale*), and negatively correlated (P < 0.05) with the difference between height at croup (*tubera sacrale*) and height at back (r = − 0.71) and hock circumference (r = − 0.77; Fig. 6). The difference between height at croup (*tubera sacrale*) and height at back was also negatively correlated with BWR_190_ (r = − 0.92, P < 0.05), and tended to be negatively correlated with BWR_La3_ (r = − 0.57, P = 0.05). *Metatarsus* circumference was positively correlated with BWR_180_ (r = 0.96, P < 0.05). The change in CK levels between rest and 24 h post exercise (post exercise minus rest) was negatively correlated with the difference between height at withers and height at back (r = − 0.52, P < 0.05) and croup (*tubera sacrale*) (r = − 0.51, P < 0.05).

### Differences in body measurements in horses that reached the lactate threshold and those that did not

Horses that reached the lactate threshold had higher BW (396 ± 7 kg) than horses that did not (357 ± 11 kg) (P < 0.05), but there were no other differences in body measurements or BCS between horses that reached the lactate threshold and those that did not (P > 0.05).

## Discussion

To our knowledge, this is the first study to examine weight carrying capacity and the relationship to conformation in Icelandic horses used by tourist companies. Overall, the physiological response of the horses to weight carrying was relatively low, with average lactate levels of 5.5 mmol/L, HR < 195 bpm at the highest load, mean BWR_La4_ > 30%, and four horses not even reaching the lactate threshold, despite carrying 35% of their BW. The response was also low compared with that observed in a previous study on Icelandic horses used for higher riding education, where eight horses reached the lactate threshold at an average BWR of ~ 23% [4]. The relatively low physiological response was unexpected, since the horses carried in total 120–160 kg in the last step (BWR_35%_), which is considerably heavier than the average weight of Scandinavian woman and men [16, 17] or of riders participating in BEFTs [1]. The horses in the present study were used to carry tourists of different body weights during long-distance tours (common distance 20–40 km/day for several days, where each horse carries a rider for approximately 7–10 km/day [18]), and compared with the Icelandic horses used for riding education studied previously [4], they presumably performed a different type of physical training and might have been physiologically and biomechanically adapted to carry heavier riders. In humans, weight carrying capacity seems to be associated with earlier experience of weight carrying [19], but no such study has been performed on horses. There are indications that Icelandic horses participating in a BEFT are pre-selected [20], and it might be the same for the horses in the current study, where the selection performed by the tour riding companies was based on qualities suitable for tour riding. A previous study showed that several body measurements differ between Icelandic horses participating in a BEFT and tour riding and school horses, but the importance of these differences and the underlying reasons are not known but were hypothesised to be due to e.g. pre-selection and training [10]. However, there was some variation in the physiological response of the horses in this study. For example, one horse was hypothesised to have reached the maximum HR at BWR_20%_ (i.e. a linear increase was not observed) and another horse reached the lactate threshold already before BWR_20%_. Similar variation in weight carrying capacity was detected in a previous study on a group of horses that had all been trained similarly [4]. The authors hypothesised that the underlying reason for the variation could be due to genetic factors [4].

As expected, the physiological response increased with increased BWR, which may in some respects be explained by an exercise duration effect. However, in the previous study [4], an additional step of BWR_20%_ was added in the end of the exercise test to investigate if the increased physiological response was due to an accumulated physiological effect or increased weight load. Plasma lactate concentration was shown to be lower in the last step compared with BWR_35%_ and HR was reduced to the same level as recorded at the first step [4], indicating that the increased physiological response was mainly due to an increased weight load.

The physiological response was similar for the two groups of horses (N and B) and no physiological parameter differed between the groups or was correlated with back score or angle of the thoracolumbar region. The angle measurements were performed in order to objectively assess back conformation, in addition to the subjective back score. Horses in group B (i.e. with broad back) had higher mean BCS compared with horses with narrow back, which was a limitation of the study since higher BCS is associated with increased physiological response in Icelandic horses [13]. However, no physiological parameter was correlated with BCS in the present study. Higher back score and lower angle of the thoracolumbar region (i.e. broader back) could be due to greater fat deposition at the thoracolumbar region, rather than a larger *m. longissimus dorsi*. However, the thoracolumbar region does not seem to be the primary location for fat deposition in moderately fleshy Icelandic horses [13]. To assess the effect of muscle mass on the back on weight carrying capacity, the size of *m. longissimus dorsi* should preferably be determined with ultrasound on horses with a similar overall BCS/body fat percentage.

Some body measurements were found to be associated with physiological parameters. For example, chest width was positively correlated with BWR_La4_. A wider chest in horses with higher BWR_La4_ may be due to that the front limbs supports the majority of the body mass during a step in tölt [21]. It has also been shown that the ground reaction force in Dutch Warmblood horse’s increases with a rider compared with trotting unmounted [22], which may indicate importance of a wide chest for weight carrying. Horses with wider chests may have a greater muscle mass in the chest area (although this was not truly measured in the present study), which in theory could be favourable for weight carrying. Lateral balance might also be enhanced with a greater distance between the front limbs. A wide chest has been shown to be favourable for riding ability in a BEFT, where the optimum width for highest subjective score for riding ability was found to be 40 cm, based on data from over 10 000 horses [11].

The difference between height at withers and height at croup (*tubera sacrale*) was positively correlated with BWR_La2_ and negatively correlated with the change in CK from rest to 24 h post exercise. In addition, there was a negative correlation between both BWR_La2_ and BWR_190_ and the difference between height at croup (*tubera sacrale*) and height at back (referred to as “back incline” in a previous study [11]). Back incline also tended to be negatively correlated with BWR_La3_. Also the difference between height at withers and height at back was negatively correlated with the change in CK. This may indicate that an “uphill” conformation and a straight backline are favourable for weight carrying capacity, at least in the Icelandic horse. An “uphill” conformation and a smaller back incline (i.e. straight backline) have been shown to be associated with higher scores for riding ability in a BEFT [11] and are rewarded in the conformation assessment [9].

In summary, conformation traits found to have the strongest correlation with a physiological parameter and/or correlated with several physiological parameters, and thus considered as the most important conformational traits for weight carrying capacity, were: a wide chest, “uphill” conformation, straight backline and smaller hock circumference. Hock circumference was collected in addition to the equivalent joint measurement of *carpus* that are made at BEFTs for Icelandic horses [9]. A too small *carpus* circumference is described as an undesired trait [9]. However, it should be noted that a correlation does not necessarily mean that there is a causality, and it is hypothesised that the correlation between chest width and BWR_La4_ have a greater causality (see discussion above) than e.g. the correlation between hock circumference and BWR_La2_. Considering the small number of horses (n = 5) in which BWR_190_ could be estimated, the importance of correlations to that parameter needs to be further investigated.

Interestingly, horses that reached the lactate threshold during the exercise test had higher BW (but not higher BCS) than horses which did not reach the lactate threshold. One possible explanation is that horses with lower BW had previously been ridden by heavier riders, relative to their own BW, and were adapted to higher BWR. This might also explain why smaller hock circumference (i.e. a smaller horse) was associated with greater weight carrying capacity. However, similar results have not been reported in previous studies on horses. Icelandic horses with higher BW have earlier been reported to show an increased physiological response during exercise [13]. However, those horses also had higher BCS and body fat percentage. In addition to BCS and body fat percentage, BW may also be affected by e.g. muscle mass and height. In humans, it has been reported that an increase in BW impair aerobic capacity (VO_2max_), regardless of whether the increase is due to an increase in body fat content or in lean body mass [23]. However, BW was not associated with any other physiological parameter in the present study.

One day after the exercise test, an additional blood sample was taken to measure plasma concentrations of muscle enzymes (AST and CK) as an indicator of unaccustomed exercise [24, 25]. Unexpectedly and in contrast to earlier findings [4], plasma AST concentration was higher before the test than at 24 h after. The reason for this is unclear, but all horses performed sub-maximal treadmill exercise for several days in a row, with the last exercise bout occurring 1–6 days before collection of the blood sample in rest. Horses with fewer days between exercise and collection of the blood sample in rest had higher values of muscle enzymes (data not shown). The training intensity on the treadmill was relatively low, but the horses were not accustomed to that type of exercise, which may have led to unusual muscle utilization and associated leakage of enzymes from muscle cells to the blood stream. It has been shown that weekly repeated exercise reduces the increase in muscle enzymes in serum after exercise [24]. The horses in the present study were used to carry riders of different weights, which may explain the lack of increased plasma muscle enzymes after the ridden exercise test. However, the resting plasma AST level (8.7 ± 0.41 µkat/L) was only slightly higher than the resting reference value range (3.1–6.2 µkat/L [26]). Hence, the difference between levels before the test and at 24 h after might not be of any biological relevance. To reduce the effect of the previous treadmill training and of individual variation, the difference in AST and CK measured before the exercise test and 24 h after was calculated and used in correlation analysis.

Limitations of this study are the number of horses included, in total and in the two groups, and that several physiological parameters could not be estimated. However, the groups were only applied in the assessment of a narrow and broad back and not in the evaluation of the effect of other body measurements.

## Conclusions

The physiological response to weight carrying in the tour riding horses used in this study was relatively low compared with earlier findings. In 27% of the horses, the lactate threshold was not reached although BWR exceeded 35%. A wider chest, “uphill” conformation, straight backline and smaller hock circumference were associated with improved weight carrying capacity (BWR_La4_ or BWR_La2_), but group (narrow or broad back) had no effect on weight carrying capacity. However, further controlled studies are needed to confirm effects of conformation in individual horses on weight carrying capacity.

## Supplementary Information

Additional file 1 (Equations and statistical codes. File format: Microsoft Word.)

## Data Availability

The datasets used and/or analysed during the current study are available from the corresponding author on reasonable request.

## Declarations

### Prior publication

Data included in this article have previously been published in the Proceedings of the 7th Animal Welfare Science Symposium, 3–4 June 2024, Uppsala, Sweden.
