# Supplementary material for: Physiological response to weight carrying and associations with conformation traits in Icelandic horses used for tour riding
Source: Acta Vet Scand. 2025 Jun 19;67:35. doi: 10.1186/s13028-025-00818-5 (PMC12180175; doi:10.1186/s13028-025-00818-5)
Supplement: Supplementary file 1 — Additional file 1 (Equations and statistical codes. File format: Microsoft Word.) [file 13028_2025_818_MOESM1_ESM.docx]

**Additional file 1** Equations and statistical codes.

**Equations**

Equation of the exponential regression between body weight ratio (BWR) and plasma lactate concentration for each step in the exercise test:

$y=a \times e^{bx}$

, where y is the plasma lactate concentration (2, 3 or 4 mmol/L), a is the lactate concentration at a BWR of 0%, b is the slope and x is the BWR at y=2, 3 or 4 mmol/L.

Equation of the linear relationship between BWR for each step and the mean heart rate (HR) of the last 15 seconds in each step:

$y=a+bx$

, where y is the HR (180 or 190 beats per minute (bpm)), a is the heart rate at a BWR of 0%, b is the slope and x is the BWR at y=180 or 190 bpm.

**Statistical codes**

Model (i):

ods graphics;

**proc** **glm**;

**PROC** **GLM** data=file PLOTS(UNPACK)=DIAGNOSTICS;

class group year sex;

model physiological parameter=group year sex bcs;

lsmeans group year sex/stderr pdiff;

means group year sex/ tukey;

**run**;

ods graphics off;

With repeated measurements:

ods graphics;

**proc** **mixed** data=file plot=(all);

class horse group sample year;

model physiological parameter=group*sample group sample year sex bcs/solution;

random horse;

repeated /subject=horse*sample type=un;

lsmeans group*sample group sample year/adjust=tukey pdiff;

**run**;

ods graphics off;

Model (ii):

ods graphics;

**proc** **glm**;

**PROC** **GLM** data=file PLOTS(UNPACK)=DIAGNOSTICS;

class group sex;

model body measurement=group sex age;

lsmeans group sex/stderr pdiff;

means group sex/ tukey;

**run**;

ods graphics off;

Model (iii):

ods graphics;

**proc** **mixed** data=file plot=(all);

class horse group sample;

model speed=group sample/solution;

random horse;

repeated /subject=horse*sample type=un;

lsmeans group sample/adjust=tukey pdiff;

**run**;

ods graphics off;
